# Tuberculosis and Its “Troubled Relationship” With Other Diseases

**DOI:** 10.7759/cureus.26482

**Published:** 2022-07-01

**Authors:** Maria João Correia, Marta Maio Herculano, Joana Duarte, Filipa Brás Monteiro, Eduarda Carmo

**Affiliations:** 1 Internal Medicine, Hospital São Francisco Xavier, Centro Hospitalar de Lisboa Ocidental, Lisbon, PRT; 2 Intensive Care Unit, Hospital Egas Moniz, Centro Hospitalar de Lisboa Ocidental, Lisbon, PRT; 3 Polyvalent Intensive Care Unit, Hospital Egas Moniz, Centro Hospitalar de Lisboa Ocidental, Lisbon, PRT

**Keywords:** urethral catheter, urethral injury, hemorrhagic shock, hiv aids, genital tuberculosis

## Abstract

Tuberculosis (TB) is a multisystemic disease caused most frequently by *Mycobacterium tuberculosis.* Extrapulmonary TB has become more frequent with the advent of human immunodeficiency virus (HIV) as HIV can facilitate the infection with *M. tuberculosis*, especially during HIV seroconversion. Here, we present the case of a 22-year-old man, from Guinea-Bissau, with a history of untreated HIV who was admitted to the intensive care unit for respiratory failure needing mechanical ventilation. Pulmonary TB was diagnosed. His stay was complicated with a hemorrhagic shock due to traumatic urethral catheterization, which led to a perforation of the capsule of the prostate. A prostatectomy was needed for bleeding control. The anatomopathological examination confirmed the presence of acid-resistant bacilli, and an extensive caseous type necrosis of the whole tissue, thus diagnosing a prostatic tuberculosis. The patient recovered after a hemorrhagic shock, a urologic and radical intervention, and some severe infectious complications.

## Introduction

Tuberculosis (TB) is a multisystemic disease caused most frequently by *Mycobacterium tuberculosis*. It can also be caused by any other organisms of the *M. tuberculosis* complex (*M. bovis*,* M. africanum*,* M. microtia*). It has many different manifestations, but the lungs are affected about 85% of the time, with TB being designated as pulmonary TB. Extrapulmonary TB has become more frequent with the advent of human immunodeficiency virus (HIV) and other immunosuppressant states, such as organ transplantation, and also with the appearance of drug-resistant mycobacteria [1].

HIV co-infection remains a real issue worldwide, and is particularly prevalent in African countries. Although TB prevalence remains high in the world, specifically in the latent stage of the disease, its global incidence is slowly declining. TB remains the most common cause of infectious disease-related mortality worldwide, and thus, it is an important public health issue [1,2].

Genitourinary TB is the third most frequent form of extrapulmonary disease, following lymph node involvement and tuberculous pleural effusion. It happens only in 2% to 20% of patients with pulmonary TB, but a higher percentage is seen when miliary disease is present (about 25%-65%) [3]. Genital TB is less common than urinary tract TB. In males, when it does occur, epididymitis is the most common finding. The prostate involvement is typically asymptomatic, occurring due to hematogenous spread [3,4].

The diagnosis of genitourinary TB is established when acid-fast bacilli are present in the urine, either by acid-fast stain, mycobacterial culture or polymerase chain reaction (PCR) for *M. tuberculosis*. The genital involvement may be suspected with suggestive clinical manifestations and relevant epidemiological factors (for instance, prior TB infection or exposure). In the absence of urinary involvement, a directed biopsy should be performed. The prostate involvement, when present, is usually an incidental finding after transurethral resection [4].

A prostate gland pathology study usually shows a nonspecific granulomatous prostatitis, but there are also other infectious diseases causing this type of nonspecific granulomatous alteration, such as *Treponema pallidum*, some viruses and fungi. In the specific case of TB, it causes a non-confined, diffuse, caseating epithelioid cell granuloma, and the Ziehl-Neelsen stain confirms this etiology [4].

## Case presentation

A 22-year-old man from Guinea-Bissau, living in Portugal for several years, with a history of non-treated HIV, was admitted to the emergency department (ED) with a three-month history of malaise, dysphonia, dry cough, pleuritic chest pain, and abdominal pain. On admission, the physical examination revealed fever (temperature 38ºC), dehydrated and icteric mucous membranes, and polypnea at rest. On pulmonary auscultation, there were decreased breath sounds bilaterally, with no other relevant findings.

Blood tests revealed pancytopenia with a normocytic normochromic anemia of 9.9 g/dL hemoglobin, leucocyte count 2200 mL/m^3^, platelets 130,000/mm, elevated C-reactive protein (CRP) level at 10.5 mg/dL, renal dysfunction (serum creatinine at 2.8 mg/dL), hepatic cytocholestasis and hyperbilirubinemia (aspartate aminotransferase 331 U/L, alanine aminotransferase 77 U/L, alkaline phosphatase 868 U/L, γ-glutamyl transferase 332 U/L, total bilirubin 3.08 mg/dL), and hyponatremia (serum sodium 123 mmol/L). The arterial blood gas analysis showed hypoxemia and compensated metabolic acidosis (pH 7.42, pCO_2_ 23.6 mmHg, pO_2_ 68.4 mmHg, HCO_3_ 15.2).

**Figure 1:**
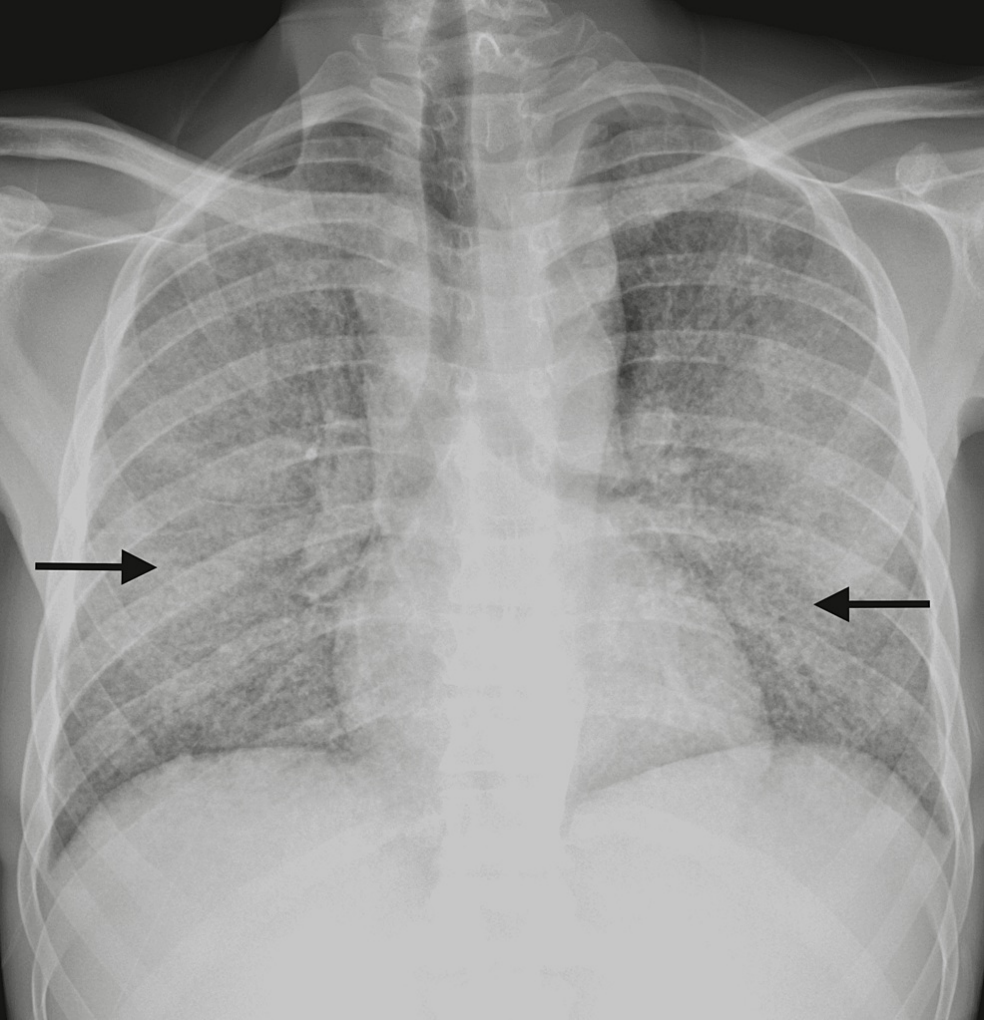
Thoracic X-ray on ICU admission The arrows show the interstitial alterations, in a bilateral diffuse distribution, which explained the clinical respiratory deterioration.

**Figure 2:**
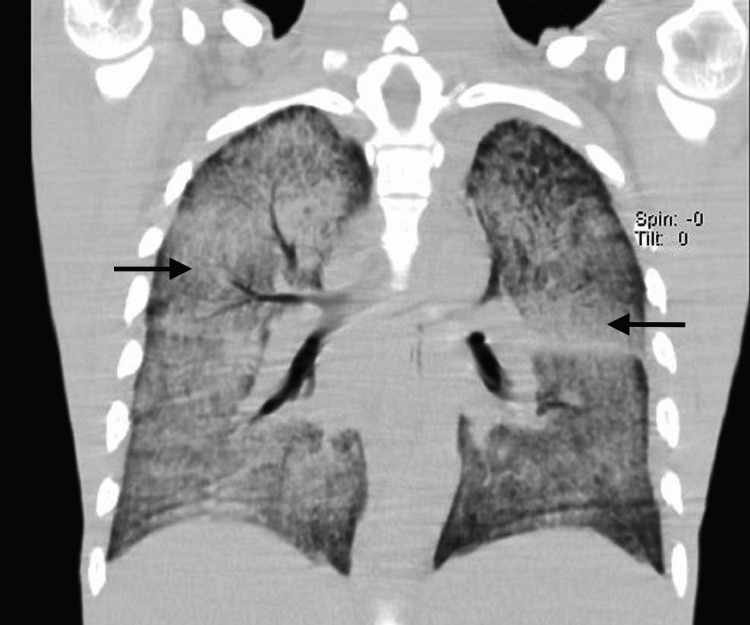
Coronal view of the thorax computed tomography scan To further investigate the alterations on the previous X-ray, a computed tomography scan was performed. As demonstrated, a severe bilateral consolidation of both lungs is present (arrows).

**Figure 3:**
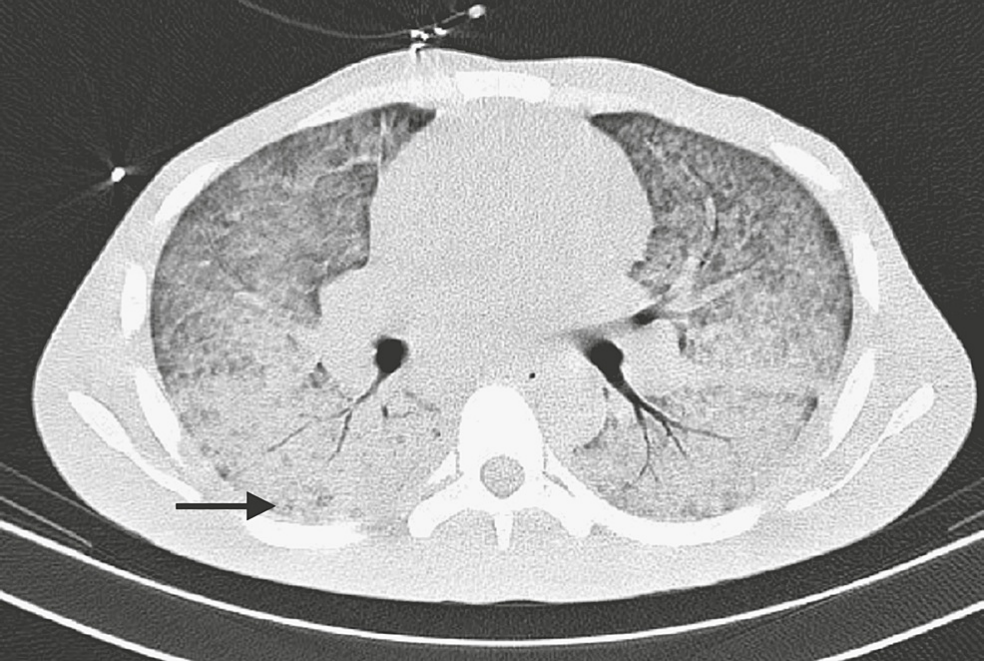
Transversal plane of the thorax computed tomography scan The arrow shows the consolidation of both lungs on ICU admission.

Chest X-ray was performed showing a diffused interstitial lung pattern. On further evaluation, the chest computed tomography revealed multiple foci of ground-glass densification, with areas of coalescence, and some consolidations with air bronchogram, also revealing large bilateral mediastinal and hilar lymph nodes (Figures 1-3). The abdominal ultrasound showed marked hepatosplenomegaly (spleen with a bipolar axis of 18.5 cm) and lumboaortic lymphadenopathies.

The increasing oxygen requirement and clinical worsening led to respiratory failure with invasive mechanical ventilation need and intensive care unit (ICU) admission.

Given his poor immunity status (HIV viral load of 6,235,502 copies/mL and a CD4+ T-lymphocyte count of 26 cell/uL), an opportunistic HIV-associated disease was considered. He was started on empiric antibiotic therapy with piperacillin-tazobactam, and cotrimoxazole for coverage of *Pneumocystis jirovecii* pneumonia.

Fiberoptic bronchoscopy was then used to perform bronchoalveolar lavage. The direct examination was positive for mycobacteria, and the cultural exam revealed methicillin-sensitive *Staphylococcus aureus*. *P. jirovecii *infection was excluded by immunofluorescence examination and PCR. Thus, co-trimoxazole was stopped (with the patient remaining on prophylactic dose); the antibiotic therapy was maintained and anti-bacillary therapy with isoniazid (H), rifampicin (R), pyrazinamide (Z) and ethambutol (E), or HRZE regimen, was started.

On the 10th day of ICU admission, macroscopic hematuria was noted. Bladder lavage was initiated promptly without any improvement, so a cystoscopy was performed, which only revealed a friable mucosa. A false path or urethral-vesical lesion was ruled out on this examination. Although continuous bladder lavage was performed, unsolved hematuria evolved into massive refractory bleeding and subsequent hemorrhagic shock. A massive transfusion protocol was activated; more than 15 packed red blood cells, 5 platelet units, 9 fresh frozen plasma, and other products, such as fibrinogen, tranexamic acid and calcium, were given in the first 24 hours of shock. High doses of vasopressors were required, with perfusion of norepinephrine up to 3 mcg/kg/min and epinephrine up to 0.5 mcg/kg/min. The patient underwent emergent urologic surgery, which confirmed a laceration of the prostatic capsule originated by a false lumen catheterization. A total prostatectomy was performed in order to control the hemorrhage. The anatomopathological examination confirmed the presence of acid-resistant bacilli, and an extensive caseous type necrosis of the whole tissue, thus diagnosing prostatic tuberculosis.

The 31-day stay of the patient in the ICU was complicated by numerous infections, namely, an abscess in the surgical site where several bacteria were isolated, requiring frequent washes and multiple courses of antibiotic therapy.

The institution of antiretroviral therapy (ART) was delayed until one month after the HRZE start, with subsequent HIV viral load control. The ART association chosen was abacavir, lamivudine and dolutegravir. The antituberculous therapy was conducted for a total of six months and a drug-susceptible TB regimen was maintained after confirming the sensitivity on prolonged cultural examination. After 43 days of HRZE, therapy was modified to levofloxacin, isoniazid and ethambutol, due to hepatotoxicity.

Although some enlarged lymph nodes were observed in many imaging studies, only pulmonary and prostatic involvement by TB were confirmed. Blood and urine cultures and PCR for mycobacteria came back negative.

**Figure 4:**
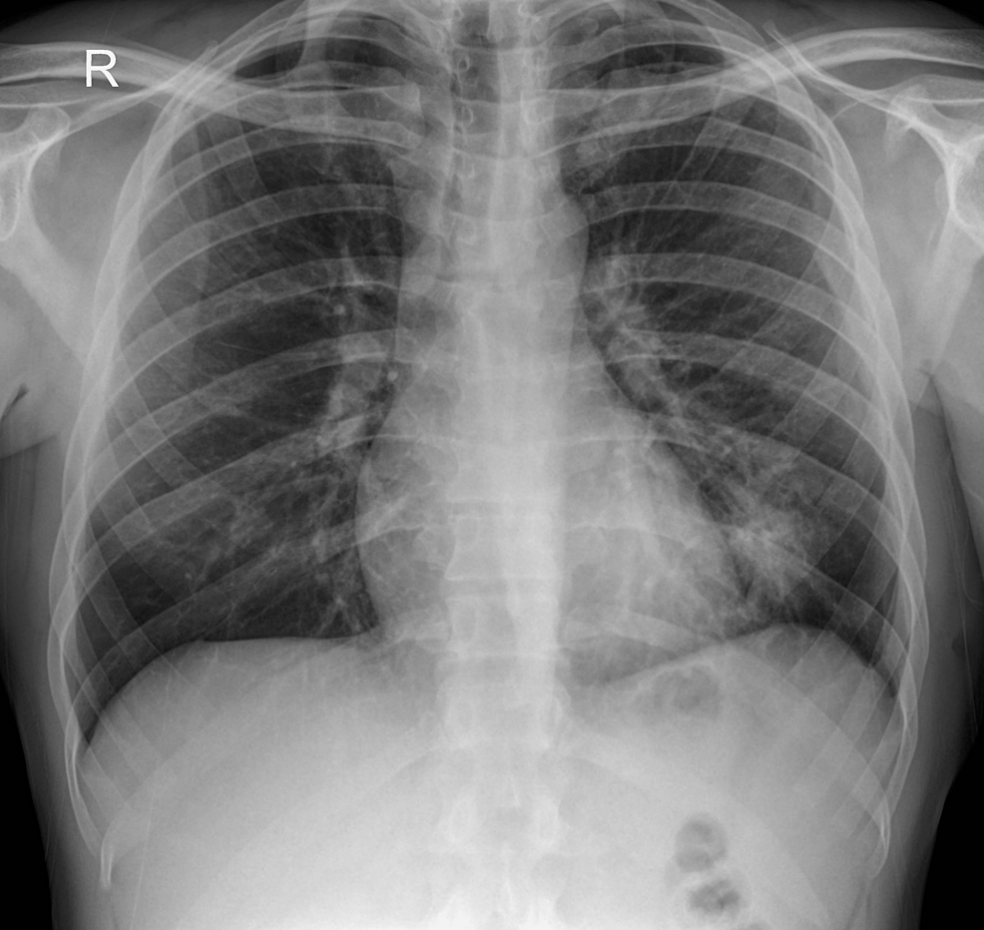
Chest X-ray after the hospital stay After two years, there seems to be no major sequel on thoracic imaging.

Two years later, the patient is still alive and he has frequent follow-ups with urology and infectious diseases consultations. He has no respiratory problems after such a long and full-of-complications hospital stay (Figure 4).

## Discussion

Although this was initially a straightforward case of pulmonary TB in an immunosuppressed patient, it quickly manifested as an interesting and unexpected presentation of prostatic TB, considering prostatic TB is a rare presentation. In this case, the HIV co-infection was an important contributor in the involvement of non-usual organs in TB infection.

HIV and TB may act synergistically, perpetuating an immunosuppressive state. HIV can facilitate the infection with *M. tuberculosis*, especially during HIV seroconversion, since it causes a rapid depletion of the T helper cells [2]. In HIV-infected patients, mycobacteria also seem to increase viral replication as measured in the peripheral circulation, lung, and lymphoid tissues, through mechanisms involving immune activation [5]. TB facilitates the replication of the virus in the cells, by activating CD4 T lymphocytes and macrophages harboring latent HIV. The onset of TB in HIV-infected patients causes a marked release of pro-inflammatory cytokines that activate lymphocytes and macrophages, which results in an increased HIV viral load [6]. There are also several studies showing that the viral load in plasma declines after the successful treatment of co-infections other than TB, but remains persistently elevated during co-infection with TB. As for the CD4 T-lymphocyte count, there is a transient CD4 lymphopenia in TB patients and a normalized CD4 T-lymphocyte count after adequate treatment [5,6].

Other concomitant infections are frequently present when there is a poor immunity status, especially when the CD4+ T-lymphocyte count is under 500 cell/uL. In this particular case, only *S. aureus* was isolated in the sputum, with all the microbiological cultures performed, and it was treated accordingly. The importance of the prophylactic measures needs to be highlighted here.

The start of ART was delayed due to the risk of immune reconstitution inflammatory syndrome, which paradoxically worsens ongoing infectious processes.

The hematologic abnormalities could also have been explained by TB. Normocytic normochromic anemia is the most common finding in TB and, even though rare, pancytopenia has been described in disseminated disease. There are several factors to be considered in the etiology of pancytopenia such as hypersplenism, maturation arrest, or bone marrow infiltration by caseating and noncaseating granulomas [7-9]. In this particular case, a bone marrow study should have been performed in order to exclude TB infiltration. There are no proper implications in this diagnosis since the treatment is the same, and a recovery of peripheral blood counts with HRZE therapy was expected.

This case also emphasizes possible complications of a procedure such as urethral catheterization, which is a simple and common medical procedure that enables the drainage of urine. It can be used for monitoring, diagnostic or therapeutic purposes. There are some complications described after the procedure, mostly harmless and common, such as urinary tract infections being the most frequent. Other known complications are genital infection, urethral bleeding, creation of a false pathway, meatal strictures and, very rarely, bladder perforation and necrosis [10]. In the presented case, the urethral catheterization caused a prostatic capsule laceration and triggered an uncontrolled hemorrhage. This iatrogenic procedure led to the finding of prostatic tuberculosis, a rare and challenging diagnosis, as described before.

## Conclusions

In the presented case report, urethral catheterization caused a prostatic capsule laceration and triggered an uncontrolled hemorrhage. This iatrogenic procedure led to the finding of prostatic tuberculosis, a rare and challenging diagnosis, as described before. This was an incident finding and it makes us think that, although a rare finding, the disseminated TB with prostate involvement is probably underdiagnosed, since it is usually an asymptomatic presentation.

The prevalence and incidence of tuberculosis in the world remains high, and its overall mortality is a real issue. Because of its different manifestations, diagnosing TB may present as a real medical challenge. Also, to emphasize that all medical procedures have associated risks, either more or less complex and frequent, we should never underestimate the interventions we conduct; iatrogenic complications happen, but should always be avoided.
